# 3-(2,3-Di­meth­oxy­phen­yl)-2,3-di­hydro-1*H*-benzo[*f*]chromen-1-one

**DOI:** 10.1107/S2414314622008859

**Published:** 2022-09-13

**Authors:** Jiha Sung

**Affiliations:** a Department of Applied Chemistry, Dongduk Women’s University, Seoul 136-714, Republic of Korea; University of Aberdeen, Scotland

**Keywords:** crystal structure, flavanone, benzochromenone, C—H⋯O hydrogen bonds

## Abstract

In the title compound, C_21_H_18_O_4_, the central pyran ring is in an envelope conformation and the dihedral angle between the benzene ring and naphthalene ring system is 88.31 (1)°. The meth­oxy groups at the *ortho* and *meta* positions of the benzene ring are tilted to the ring with C—C—O—C torsion angles of 105.9 (4) and 9.5 (5)°, respectively. In the crystal, pairwise C—H⋯O hydrogen bonds form *R* ^2^_2_(14) inversion dimers, which are linked by another pair of C—H⋯O hydrogen bonds to form [210] chains in the crystal.

## Abstract 2

The crystal structure of a flavanone is reported in which C—H⋯O hydrogen bonds link the mol­ecules into chains.

## Graphical abstract

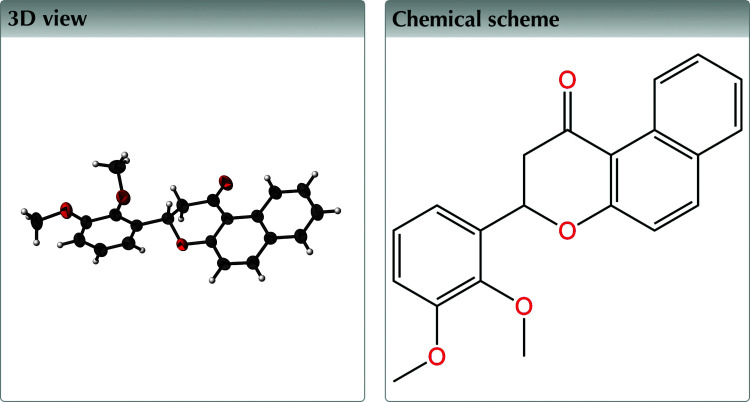

## Structure description

Flavanones exhibit a wide range of biological properties, including anti­viral (Shi *et al.*, 2022 ▸), anti­fungal (Emami *et al.* 2013 ▸) and anti­cancer activities (Bailly, 2021 ▸; Zhao *et al.*, 2019 ▸) as well as being used in the treatment of Alzheimer’s disease (Jin *et al.*, 2021 ▸). In continuation of our research into flavanone derivatives (Sung, 2020 ▸), the title compound was synthesized and its crystal structure was determined.

**Figure 1:**
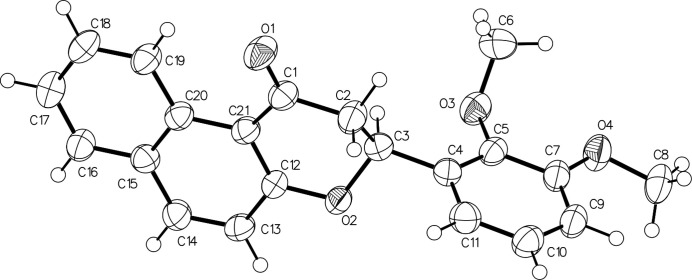
The mol­ecular structure of the title compound, showing the atom-labelling scheme and displacement ellipsoids drawn at the 50% probability level.

**Figure 2:**
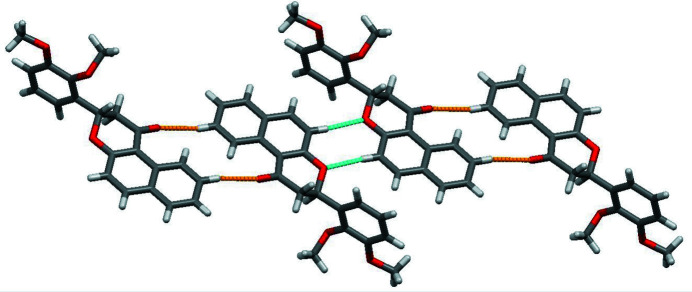
Part of the crystal structure of the title compound, showing the weak C—H⋯O hydrogen bonds forming 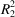 (14) dimers as yellow lines. An additional pair of inter­molecular hydrogen bonds (blue lines) link the dimers to form a chain.

The title compound, C_21_H_18_O_4_, was prepared in a two-step reaction. A Claisen–Schmidt condensation reaction between 2,3-dimeth­oxy-benzaldehyde and 2-hy­droxy-1-aceto­naphthone gave the corresponding benzochalcone, which was then used for an intra­molecular Michael addition reaction to provide the desired flavanone (Yong *et al.* 2014 ▸). The mol­ecular structure of the title compound is shown in Fig. 1 ▸. The central pyran ring (C1/C2/C3/O2/C12/C21) has an envelope conformation with atom C3 as the flap. C3 is a stereogenic centre: in the arbitrarily chosen asymmetric unit, C3 has an *S* configuration, but crystal symmetry generates a racemic mixture. The hydrogen atom H3 attached to C3 forms a *trans* diaxial conformation with atom H2*B* of the C2 methyl­ene group (H3—C3—C2—H2*B* = −179.1°) and a *gauche* conformation with the other H atom attached to C2 (H3—C3—C2—H2*A* = −60.8°). The meth­oxy group at the *meta* position of the benzene ring is twisted slightly from the ring [C9—C7—O4—C8 = 9.5 (5)°]. However, the meth­oxy group at the *ortho* position is significantly distorted from the benzene ring due to steric hindrance with the pyran ring [C4—C5—O3—C6 = 105.9 (4)°]. The C12–C21 naphthalene ring system (r.m.s. deviation = 0.036 Å) and benzene ring (C4/ C5/C7/ C11/C9/C10]; r.m.s. deviation = 0.003 Å) lie almost perpendicular to each other forming a dihedral angle of 88.31 (1)°. In the crystal, pairs of C18—H18⋯O1 hydrogen bonds form an inversion dimer with graph-set notation 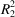 (14). The dimers are linked by another pair of C13—H13⋯O2 hydrogen bonds to form a [210] chain. (Table 1 ▸, Fig. 2 ▸).

## Synthesis and crystallization

A solution of 2-hy­droxy-1-aceto­naphthone (186 mg, 1 mmol) and 2,3-di­meth­oxy­benzaldehyde (166 mg, 1 mmol) was dissolved in ethanol (15 ml) and the temperature was adjusted to around 276–277 K in an ice bath. To the cooled reaction mixture was added 1.0 ml of 40% aqueous KOH solution, and the reaction mixture was stirred at room temperature for 24 h. This mixture was poured into iced water (50 ml) acidified with 6 *N* HCl solution. The mixture was extracted with ethyl acetate (3 × 30 ml) and the combined organic layers were dried under MgSO_4_. Filtration and evaporation of the filtrate gave a solid chalcone, which was dissolved in DMSO and a catalytic amount of conc. HCl was added. After 10 h, the reaction mixture was poured into iced water to give a solid flavanone. Recrystallization from ethanol solution gave the crystals used in this X-ray diffraction study.

## Refinement

Crystal data, data collection and structure refinement details are summarized in Table 2 ▸.

## Supplementary Material

Crystal structure: contains datablock(s) I. DOI: 10.1107/S2414314622008859/hb4411sup1.cif

Structure factors: contains datablock(s) I. DOI: 10.1107/S2414314622008859/hb4411Isup2.hkl

CCDC reference: 2205278

Additional supporting information:  crystallographic information; 3D view; checkCIF report

## Figures and Tables

**Table 1:** Hydrogen-bond geometry (Å, °)

*D*—H⋯*A*	*D*—H	H⋯*A*	*D*⋯*A*	*D*—H⋯*A*
C13—H13⋯O2^i^	0.95	2.52	3.454 (4)	169
C18—H18⋯O1^ii^	0.95	2.52	3.452 (4)	166

Symmetry codes: (i) 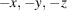 ; (ii) 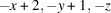 .

**Table 2:** Experimental details

Crystal data	
Chemical formula	C_21_H_18_O_4_
*M*_r_	334.35
Crystal system, space group	Triclinic, *P* 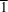
Temperature (K)	200
*a*, *b*, *c* (Å)	8.3312 (14), 9.6506 (16), 11.797 (2)
α, β, γ (°)	94.261 (4), 107.335 (4), 112.326 (3)
*V* (Å^3^)	818.4 (2)
*Z*	2
Radiation type	Mo *K*α
μ (mm^−1^)	0.09
Crystal size (mm)	0.34 × 0.21 × 0.16

Data collection	
Diffractometer	Bruker *SMART* CCD
Absorption correction	Multi-scan (*SADABS*; Krause *et al.*, 2015 ▸)
*T*_min_, *T*_max_	0.969, 0.985
No. of measured, independent and observed [*I* > 2σ(*I*)] reflections	5181, 3202, 2136
*R*_int_	0.020
(sin θ/λ)_max_ (Å^−1^)	0.618

Refinement	
*R*[*F* ^2^ > 2σ(*F* ^2^)], *wR*(*F* ^2^), *S*	0.057, 0.204, 1.13
No. of reflections	3202
No. of parameters	228
H-atom treatment	H-atom parameters constrained
Δρ_max_, Δρ_min_ (e Å^−3^)	0.30, −0.38

Computer programs: *APEX2* and *SAINT* (Bruker, 2012 ▸), *SHELXS* and *SHELXTL* (Sheldrick, 2008 ▸) , *SHELXL2014* (Sheldrick, 2015 ▸) and *publCIF* (Westrip, 2010 ▸).

## full crystallographic data

### Crystal data

**Table:**

C_21_H_18_O_4_	*Z* = 2
*M**_r_* = 334.35	*F*(000) = 352
Triclinic, *P*1	*D*_x_ = 1.357 Mg m^−^^3^
Hall symbol: -P 1	Mo *K*α radiation, λ = 0.71073 Å
*a* = 8.3312 (14) Å	Cell parameters from 2013 reflections
*b* = 9.6506 (16) Å	θ = 2.7–25.9°
*c* = 11.797 (2) Å	µ = 0.09 mm^−^^1^
α = 94.261 (4)°	*T* = 200 K
β = 107.335 (4)°	Block, yellow
γ = 112.326 (3)°	0.34 × 0.21 × 0.16 mm
*V* = 818.4 (2) Å^3^	

### Data collection

**Table:**

Bruker SMART CCD diffractometer	3202 independent reflections
Radiation source: fine-focus sealed tube	2136 reflections with *I* > 2σ(*I*)
Graphite monochromator	*R*_int_ = 0.020
ω scans	θ_max_ = 26.1°, θ_min_ = 2.3°
Absorption correction: multi-scan (SADABS; Krause *et al.*, 2015)	*h* = −10→9
*T*_min_ = 0.969, *T*_max_ = 0.985	*k* = −11→11
5181 measured reflections	*l* = −14→13

### Refinement

**Table:**

Refinement on *F*^2^	Primary atom site location: structure-invariant direct methods
Least-squares matrix: full	Secondary atom site location: difference Fourier map
*R*[*F*^2^ > 2σ(*F*^2^)] = 0.057	Hydrogen site location: inferred from neighbouring sites
*wR*(*F*^2^) = 0.204	H-atom parameters constrained
*S* = 1.13	*w* = 1/[σ^2^(*F*_o_^2^) + (0.0727*P*)^2^ + 0.8943*P*] where *P* = (*F*_o_^2^ + 2*F*_c_^2^)/3
3202 reflections	(Δ/σ)_max_ < 0.001
228 parameters	Δρ_max_ = 0.30 e Å^−^^3^
0 restraints	Δρ_min_ = −0.38 e Å^−^^3^

### Special details

**Table:**

Geometry. All esds (except the esd in the dihedral angle between two l.s. planes) are estimated using the full covariance matrix. The cell esds are taken into account individually in the estimation of esds in distances, angles and torsion angles; correlations between esds in cell parameters are only used when they are defined by crystal symmetry. An approximate (isotropic) treatment of cell esds is used for estimating esds involving l.s. planes.
Refinement. Refinement of F^2^ against ALL reflections. The weighted R-factor wR and goodness of fit S are based on F^2^, conventional R-factors R are based on F, with F set to zero for negative F^2^. The threshold expression of F^2^ > 2sigma(F^2^) is used only for calculating R-factors(gt) etc. and is not relevant to the choice of reflections for refinement. R-factors based on F^2^ are statistically about twice as large as those based on F, and R- factors based on ALL data will be even larger.

### Fractional atomic coordinates and isotropic or equivalent isotropic displacement parameters (Å^2^)

**Table:**

	*x*	*y*	*z*	*U*_iso_*/*U*_eq_	
C1	0.5916 (4)	0.4265 (4)	0.1200 (3)	0.0387 (7)	
O1	0.7393 (3)	0.5280 (3)	0.1255 (2)	0.0534 (7)	
C2	0.4897 (5)	0.4555 (4)	0.1987 (3)	0.0467 (8)	
H2A	0.5804	0.5215	0.2782	0.056*	
H2B	0.4162	0.5101	0.1594	0.056*	
C3	0.3641 (4)	0.3088 (4)	0.2179 (3)	0.0404 (8)	
H3	0.4419	0.2569	0.2588	0.048*	
O2	0.2359 (3)	0.2101 (2)	0.10246 (19)	0.0406 (6)	
C4	0.2501 (4)	0.3214 (4)	0.2935 (3)	0.0387 (7)	
C5	0.2559 (4)	0.2556 (3)	0.3936 (3)	0.0368 (7)	
O3	0.3634 (3)	0.1743 (3)	0.4229 (2)	0.0470 (6)	
C6	0.5292 (5)	0.2525 (5)	0.5253 (4)	0.0649 (11)	
H6A	0.6015	0.3530	0.5121	0.097*	
H6B	0.6025	0.1923	0.5364	0.097*	
H6C	0.4983	0.2664	0.5979	0.097*	
C7	0.1468 (4)	0.2610 (4)	0.4626 (3)	0.0396 (7)	
O4	0.1617 (3)	0.1893 (3)	0.5578 (2)	0.0501 (6)	
C8	0.0315 (5)	0.1696 (5)	0.6180 (4)	0.0558 (10)	
H8A	0.0463	0.2702	0.6546	0.084*	
H8B	0.0537	0.1122	0.6816	0.084*	
H8C	−0.0947	0.1128	0.5590	0.084*	
C9	0.0321 (5)	0.3356 (4)	0.4296 (3)	0.0443 (8)	
H9	−0.0429	0.3403	0.4753	0.053*	
C10	0.0275 (5)	0.4029 (4)	0.3300 (3)	0.0474 (8)	
H10	−0.0501	0.4549	0.3083	0.057*	
C11	0.1337 (5)	0.3958 (4)	0.2616 (3)	0.0452 (8)	
H11	0.1277	0.4416	0.1928	0.054*	
C12	0.3185 (4)	0.1820 (4)	0.0248 (3)	0.0366 (7)	
C13	0.2063 (4)	0.0489 (4)	−0.0672 (3)	0.0425 (8)	
H13	0.0818	−0.0104	−0.0740	0.051*	
C14	0.2764 (5)	0.0057 (4)	−0.1459 (3)	0.0427 (8)	
H14	0.1997	−0.0838	−0.2083	0.051*	
C15	0.4630 (4)	0.0918 (4)	−0.1370 (3)	0.0378 (7)	
C16	0.5352 (5)	0.0391 (4)	−0.2161 (3)	0.0428 (8)	
H16	0.4571	−0.0510	−0.2778	0.051*	
C17	0.7169 (5)	0.1166 (4)	−0.2048 (3)	0.0482 (9)	
H17	0.7653	0.0800	−0.2577	0.058*	
C18	0.8306 (5)	0.2500 (4)	−0.1150 (3)	0.0474 (9)	
H18	0.9572	0.3027	−0.1065	0.057*	
C19	0.7633 (5)	0.3060 (4)	−0.0389 (3)	0.0438 (8)	
H19	0.8427	0.3985	0.0200	0.053*	
C20	0.5757 (4)	0.2274 (4)	−0.0469 (3)	0.0366 (7)	
C21	0.4989 (4)	0.2770 (3)	0.0347 (3)	0.0364 (7)	

### Atomic displacement parameters (Å^2^)

**Table:**

	*U*^11^	*U*^22^	*U*^33^	*U*^12^	*U*^13^	*U*^23^
C1	0.0354 (17)	0.0354 (16)	0.0444 (18)	0.0114 (14)	0.0171 (14)	0.0101 (14)
O1	0.0415 (14)	0.0415 (13)	0.0690 (17)	0.0044 (11)	0.0286 (13)	0.0005 (12)
C2	0.0425 (19)	0.0418 (18)	0.053 (2)	0.0108 (15)	0.0244 (16)	−0.0009 (16)
C3	0.0327 (16)	0.0440 (18)	0.0390 (17)	0.0112 (14)	0.0130 (14)	0.0041 (14)
O2	0.0309 (11)	0.0458 (12)	0.0404 (12)	0.0101 (10)	0.0165 (10)	0.0001 (10)
C4	0.0335 (16)	0.0407 (17)	0.0353 (17)	0.0101 (14)	0.0120 (14)	0.0028 (14)
C5	0.0330 (16)	0.0334 (16)	0.0428 (18)	0.0123 (13)	0.0157 (14)	0.0027 (14)
O3	0.0464 (14)	0.0478 (13)	0.0530 (14)	0.0236 (11)	0.0217 (12)	0.0090 (11)
C6	0.048 (2)	0.087 (3)	0.055 (2)	0.034 (2)	0.0076 (19)	0.006 (2)
C7	0.0399 (18)	0.0389 (17)	0.0393 (17)	0.0124 (15)	0.0198 (15)	0.0042 (14)
O4	0.0556 (15)	0.0598 (15)	0.0489 (14)	0.0277 (13)	0.0312 (12)	0.0186 (12)
C8	0.061 (2)	0.061 (2)	0.056 (2)	0.023 (2)	0.039 (2)	0.0157 (19)
C9	0.0443 (19)	0.0452 (18)	0.0471 (19)	0.0172 (16)	0.0246 (16)	0.0045 (15)
C10	0.0438 (19)	0.050 (2)	0.053 (2)	0.0234 (17)	0.0196 (17)	0.0080 (17)
C11	0.0451 (19)	0.0470 (19)	0.0431 (19)	0.0180 (16)	0.0163 (16)	0.0131 (15)
C12	0.0325 (16)	0.0436 (17)	0.0360 (17)	0.0157 (14)	0.0158 (14)	0.0080 (14)
C13	0.0292 (16)	0.0429 (18)	0.0457 (19)	0.0065 (14)	0.0136 (14)	0.0007 (15)
C14	0.0402 (18)	0.0417 (18)	0.0411 (18)	0.0120 (15)	0.0161 (15)	0.0013 (14)
C15	0.0361 (17)	0.0385 (17)	0.0371 (17)	0.0141 (14)	0.0127 (14)	0.0084 (14)
C16	0.0433 (19)	0.0439 (18)	0.0452 (19)	0.0190 (15)	0.0208 (16)	0.0074 (15)
C17	0.054 (2)	0.050 (2)	0.053 (2)	0.0255 (18)	0.0306 (18)	0.0131 (17)
C18	0.0397 (18)	0.050 (2)	0.061 (2)	0.0178 (16)	0.0294 (17)	0.0189 (17)
C19	0.0370 (18)	0.0438 (18)	0.051 (2)	0.0136 (15)	0.0213 (16)	0.0105 (15)
C20	0.0343 (16)	0.0380 (16)	0.0404 (17)	0.0147 (14)	0.0165 (14)	0.0135 (14)
C21	0.0315 (16)	0.0371 (16)	0.0396 (17)	0.0120 (13)	0.0143 (14)	0.0080 (14)

### Geometric parameters (Å, º)

**Table:**

C1—O1	1.227 (4)	C8—H8C	0.9800
C1—C21	1.471 (4)	C9—C10	1.381 (5)
C1—C2	1.507 (4)	C9—H9	0.9500
C2—C3	1.488 (5)	C10—C11	1.379 (5)
C2—H2A	0.9900	C10—H10	0.9500
C2—H2B	0.9900	C11—H11	0.9500
C3—O2	1.439 (4)	C12—C21	1.394 (4)
C3—C4	1.513 (4)	C12—C13	1.406 (4)
C3—H3	1.0000	C13—C14	1.352 (4)
O2—C12	1.366 (3)	C13—H13	0.9500
C4—C5	1.379 (4)	C14—C15	1.423 (4)
C4—C11	1.396 (5)	C14—H14	0.9500
C5—O3	1.389 (4)	C15—C20	1.410 (4)
C5—C7	1.400 (4)	C15—C16	1.410 (4)
O3—C6	1.419 (4)	C16—C17	1.369 (5)
C6—H6A	0.9800	C16—H16	0.9500
C6—H6B	0.9800	C17—C18	1.396 (5)
C6—H6C	0.9800	C17—H17	0.9500
C7—O4	1.362 (4)	C18—C19	1.367 (5)
C7—C9	1.388 (5)	C18—H18	0.9500
O4—C8	1.428 (4)	C19—C20	1.424 (4)
C8—H8A	0.9800	C19—H19	0.9500
C8—H8B	0.9800	C20—C21	1.449 (4)
			
O1—C1—C21	123.8 (3)	C10—C9—C7	119.7 (3)
O1—C1—C2	119.8 (3)	C10—C9—H9	120.1
C21—C1—C2	116.4 (3)	C7—C9—H9	120.1
C3—C2—C1	110.8 (3)	C11—C10—C9	121.1 (3)
C3—C2—H2A	109.5	C11—C10—H10	119.5
C1—C2—H2A	109.5	C9—C10—H10	119.5
C3—C2—H2B	109.5	C10—C11—C4	120.0 (3)
C1—C2—H2B	109.5	C10—C11—H11	120.0
H2A—C2—H2B	108.1	C4—C11—H11	120.0
O2—C3—C2	109.8 (3)	O2—C12—C21	123.3 (3)
O2—C3—C4	107.0 (2)	O2—C12—C13	114.5 (3)
C2—C3—C4	116.2 (3)	C21—C12—C13	122.3 (3)
O2—C3—H3	107.8	C14—C13—C12	119.7 (3)
C2—C3—H3	107.8	C14—C13—H13	120.2
C4—C3—H3	107.8	C12—C13—H13	120.2
C12—O2—C3	114.2 (2)	C13—C14—C15	121.3 (3)
C5—C4—C11	118.8 (3)	C13—C14—H14	119.4
C5—C4—C3	120.3 (3)	C15—C14—H14	119.4
C11—C4—C3	120.9 (3)	C20—C15—C16	120.5 (3)
C4—C5—O3	119.6 (3)	C20—C15—C14	119.6 (3)
C4—C5—C7	121.4 (3)	C16—C15—C14	119.9 (3)
O3—C5—C7	118.9 (3)	C17—C16—C15	120.6 (3)
C5—O3—C6	114.0 (3)	C17—C16—H16	119.7
O3—C6—H6A	109.5	C15—C16—H16	119.7
O3—C6—H6B	109.5	C16—C17—C18	119.5 (3)
H6A—C6—H6B	109.5	C16—C17—H17	120.2
O3—C6—H6C	109.5	C18—C17—H17	120.2
H6A—C6—H6C	109.5	C19—C18—C17	121.2 (3)
H6B—C6—H6C	109.5	C19—C18—H18	119.4
O4—C7—C9	125.0 (3)	C17—C18—H18	119.4
O4—C7—C5	116.0 (3)	C18—C19—C20	120.8 (3)
C9—C7—C5	119.0 (3)	C18—C19—H19	119.6
C7—O4—C8	117.8 (3)	C20—C19—H19	119.6
O4—C8—H8A	109.5	C15—C20—C19	117.4 (3)
O4—C8—H8B	109.5	C15—C20—C21	119.2 (3)
H8A—C8—H8B	109.5	C19—C20—C21	123.4 (3)
O4—C8—H8C	109.5	C12—C21—C20	117.7 (3)
H8A—C8—H8C	109.5	C12—C21—C1	117.6 (3)
H8B—C8—H8C	109.5	C20—C21—C1	124.5 (3)
			
O1—C1—C2—C3	−156.5 (3)	C3—O2—C12—C13	158.7 (3)
C21—C1—C2—C3	26.8 (4)	O2—C12—C13—C14	−176.9 (3)
C1—C2—C3—O2	−57.2 (4)	C21—C12—C13—C14	3.3 (5)
C1—C2—C3—C4	−178.8 (3)	C12—C13—C14—C15	0.6 (5)
C2—C3—O2—C12	55.5 (3)	C13—C14—C15—C20	−2.0 (5)
C4—C3—O2—C12	−177.5 (3)	C13—C14—C15—C16	176.4 (3)
O2—C3—C4—C5	111.0 (3)	C20—C15—C16—C17	1.6 (5)
C2—C3—C4—C5	−125.9 (3)	C14—C15—C16—C17	−176.8 (3)
O2—C3—C4—C11	−67.2 (4)	C15—C16—C17—C18	−0.8 (5)
C2—C3—C4—C11	55.9 (4)	C16—C17—C18—C19	−0.9 (5)
C11—C4—C5—O3	176.6 (3)	C17—C18—C19—C20	1.7 (5)
C3—C4—C5—O3	−1.6 (4)	C16—C15—C20—C19	−0.8 (5)
C11—C4—C5—C7	0.5 (5)	C14—C15—C20—C19	177.6 (3)
C3—C4—C5—C7	−177.7 (3)	C16—C15—C20—C21	−178.7 (3)
C4—C5—O3—C6	105.9 (4)	C14—C15—C20—C21	−0.3 (5)
C7—C5—O3—C6	−77.9 (4)	C18—C19—C20—C15	−0.9 (5)
C4—C5—C7—O4	178.7 (3)	C18—C19—C20—C21	177.0 (3)
O3—C5—C7—O4	2.6 (4)	O2—C12—C21—C20	174.8 (3)
C4—C5—C7—C9	−0.6 (5)	C13—C12—C21—C20	−5.5 (5)
O3—C5—C7—C9	−176.7 (3)	O2—C12—C21—C1	−10.3 (5)
C9—C7—O4—C8	9.5 (5)	C13—C12—C21—C1	169.4 (3)
C5—C7—O4—C8	−169.8 (3)	C15—C20—C21—C12	3.9 (4)
O4—C7—C9—C10	−179.3 (3)	C19—C20—C21—C12	−173.9 (3)
C5—C7—C9—C10	0.0 (5)	C15—C20—C21—C1	−170.6 (3)
C7—C9—C10—C11	0.7 (5)	C19—C20—C21—C1	11.6 (5)
C9—C10—C11—C4	−0.8 (5)	O1—C1—C21—C12	−170.1 (3)
C5—C4—C11—C10	0.2 (5)	C2—C1—C21—C12	6.4 (4)
C3—C4—C11—C10	178.4 (3)	O1—C1—C21—C20	4.4 (5)
C3—O2—C12—C21	−21.5 (4)	C2—C1—C21—C20	−179.1 (3)

### Hydrogen-bond geometry (Å, º)

**Table:**

*D*—H···*A*	*D*—H	H···*A*	*D*···*A*	*D*—H···*A*
C13—H13···O2^i^	0.95	2.52	3.454 (4)	169
C18—H18···O1^ii^	0.95	2.52	3.452 (4)	166

Symmetry codes: (i) −*x*, −*y*, −*z*; (ii) −*x*+2, −*y*+1, −*z*.
